# Relationship between diet-related inflammation and bone health under different levels of body mass index

**DOI:** 10.1186/s13018-022-03481-y

**Published:** 2023-01-02

**Authors:** Guixing Zeng, Xiaoting Chen, Ziyan Jiang, Jiarong Lin, Yuchi Wu, Junping Wei

**Affiliations:** 1 Guang’anmen Hospital, China Academy of Chinese Medical Sciences, Beijing, 100053 China; 2 Second Affiliated Hospital, Guangzhou University of Chinese Medicine (Guangdong Provincial Hospital of Chinese Medicine), Guangzhou, 510120 China; 3 First Affiliated Hospital, Guangzhou University of Chinese Medicine, Guangzhou, 510080 China

**Keywords:** BMI, Dietary inflammatory index, Joint influence, Bone health, National Health and Nutrition Examination Survey

## Abstract

**Background:**

Osteoporosis is a major public health problem. Dietary inflammatory preference and body mass index (BMI) are emerging factors that tends to affect bone health. There is limited evidence regarding the joint influence of BMI and dietary status on the bone health. This study aimed to investigate the relationship between dietary inflammatory index (DII) and bone health among adults under different levels of BMI utilizing the National Health and Nutrition Examination Survey (NHANES).

**Methods:**

Data were collected from 2005–2010, 2013–2014 to 2017–2018 in NHANES. In total, 10,521 participants who aged ≥ 20 years and had complete data for dietary intake interview, bone mineral density (BMD) and bone mineral content (BMC) were included. DII was performed to evaluate the dietary inflammatory potential based on dietary intake interview. We evaluated bone health by femoral neck BMD and BMC measured by dual energy X-ray absorptiometry. Weighted multivariable linear regression and BMI-stratified subgroup analysis were performed.

**Results:**

The average DII score for 10,521 participants was 1.24 ± 0.04, mean femoral neck BMD was 0.82 ± 0.00 g/cm^2^ and mean BMC was 4.37 ± 0.01 g. In the fully adjusted model, there was a negative correlation between DII with BMD (*β* = − 0.016, *P* < 0.001) and BMC (*β* = − 0.011, *P* < 0.001) in the most anti-inflammatory diet. Using BMI-stratified subgroup analysis, this correlation became more evident in both the overweight (BMD: *β* = − 0.024, *P* < 0.001; BMC: *β* = − 0.058, *P* = 0.042) and obese groups (BMD: *β* = − 0.015, *P* = 0.049; BMC: *β* = − 0.009, *P* = 0.042), while this correlation was opposite in DII tertile 2 (middle DII score) in the underweight group (BMD: *β* = 0.047, *P* = 0.038; BMC: *β* = 0.274, *P* = 0.010).

**Conclusion:**

Relationship between higher consumption of pro-inflammatory and increased risk of lower BMD and BMC was only existed in overweight and obese participants.

## Introduction

Osteoporosis is one of the most common public health problems worldwide, characterized by bone loss, degeneration of bone tissue, and destruction of bone microarchitecture, resulting in a higher risk of fractures and a significant financial burden [1, 2]. It was reported that in the United States, one-third of adult females and one-fifth adult males will experience osteoporotic fracture [3]. The prevalence of osteoporosis is continuing to increase with the global population ageing.

A growing of evidence has reported that inflammation is connected to bone health [4, 5]. Several clinical studies revealed that there was inverse relationship between bone mineral density (BMD) and inflammatory markers, including C-reactive protein [6] and neutrophil lymphocyte ratio [7]. Additionally, the important role of nuclear factor kappa B (NF-kB) in the progressive process of bone has been verified in NFkB1 and NFkB2 knockout mice [8]. The role of other pro-inflammatory cytokines in bone loss has also been demonstrated using a number of experiments in vitro [9–12].

The intake of pro-inflammatory diet may play an important role in bone heath. A number of recent systematic reviews and meta-analyses reported that dietary behaviors involving higher consumption of vitamin C, vitamin D, calcium, zinc and *n*-3 polyunsaturated fatty acid were linked with low incidence of bone loss [13–15]. Instead of focused on specific nutrients or dietary patterns, dietary inflammatory index (DII), a literature-based scoring algorithm based on the effort of 45 different food components on inflammatory biomarkers, was focused on quantitating the overall inflammatory potential of individual diets [16]. A higher DII score was independently linked with obese, malnutrition–inflammation status and cardiovascular disease [17–19].

There is still controversy whether higher body mass index (BMI) is a risk or protective factor for bone heath. A systematic review and meta-analysis reported that obese and overweight patients were found to have lower risk of vertebral fractures [20]. However, a population-based prospective study based on 60,393 women indicated that obesity was a risk factor for fracture in postmenopausal women [21, 22]. Therefore, determining the joint influence of dietary inflammatory potential and BMI on the bone health might provide information for the prevention and treatment of osteoporosis. In this study, the data of 10,521 participants were extracted from NHANES to determine the association between diet-related inflammation and bone health, especially based on the BMI-stratified subgroup analysis.

## Materials and methods

### Study population

All data in this study were obtained from the National Health and Nutrition Examination Survey (NHANES). NHANES is a widespread and continuing cross-sectional program and conducted to assess individuals’ health and nutrition status in the United States by collecting interviews, physical examinations and laboratory tests. All participants provided written informed consent. All data in this study are publicly accessible at http://www.cdc.gov/nchs/nhanes/.

**Fig. 1:**
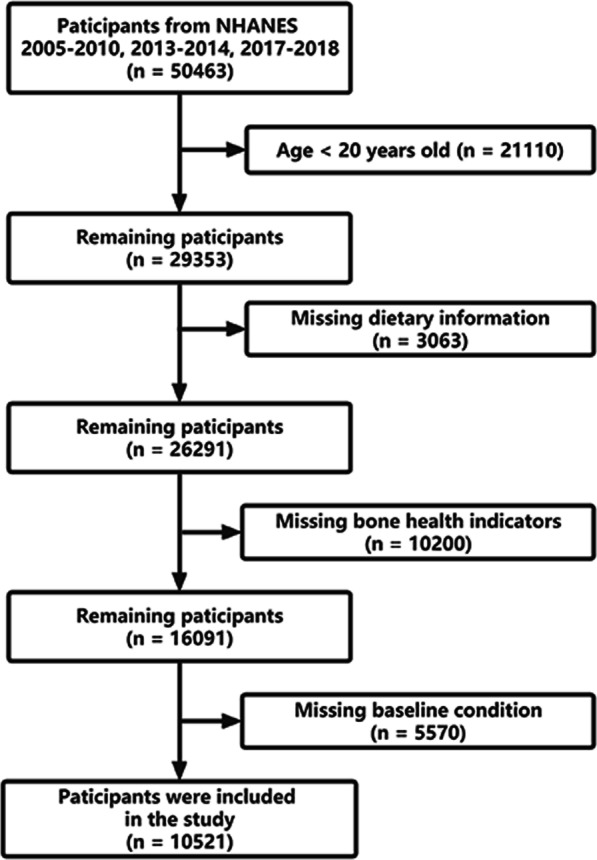
Study flowchart. NHANES, National Health and Nutrition Examination Survey

This study was based on data from 2005–2006, 2007–2008, 2009–2010, 2013–2014 to 2017–2018 in NHANES. As shown in Fig. 1, a total of 50,463 participants were involved. Of these participants, 39942 were excluded based on the exclusion of individuals age < 20 years old (*n* = 2110), missing dietary information (*n* = 3063), missing bone health indicators (*n* = 10,200) and missing baseline condition (*n* = 5570). 10,521 participants were enrolled in the final analysis.

### Exposure variable

Dietary inflammatory index (DII), a literature-based scoring algorithm based on the effort of 45 different food components on inflammatory biomarkers, has been constructed to quantitate the overall inflammatory potential of individual diets [16]. 24-h dietary recalls were utilized to calculate individual dietary intakes, which have been examined by the Nutrition Methodology Working Group [23]. To ensure precise food recall and alleviate the respondent burden, eligibility criteria included subjects who had both two valid 24-h dietary recalls.

A total of 28 food parameters were utilized to calculate the DII score, including carbohydrates, protein, total fat, alcohol, fibre, cholesterol, saturated fat, monounsaturated fatty acid (MUFA), polyunsaturated fatty acids (PUFA), *n*-3 fatty acids, *n*-6 fatty acids, niacin, vitamin A, thiamin (vitamin B1), riboflavin (vitamin B2), vitamin B6, vitamin B12, vitamin C, vitamin D, vitamin E, Fe, Mg, zinc, selenium, folic acid, beta-carotene, caffeine and energy. Previous study showed that using 28 components did not influence the DII predictive capacity [24]. Details of the DII scoring algorithm have been described previously [19]. First, global daily mean intake was adopted to calculate every participant’s *Z* score. Next, every individual *Z* score was converted to a central percentile. Finally, the overall inflammatory effort score was utilized to multiply every central percentile and then summed to gain each participant’s DII score. Higher positive DII scores represented more pro-inflammatory diets, while more negative DII scores consistent with more anti-inflammatory diet.

### Outcome variable

The outcome of interest was bone health, which was estimated by femoral neck BMD and bone mineral content (BMC). In this study, the femoral neck BMD and BMC were employed to analyzed due to its highest predictive value for hip fracture, and the hip is the site of high clinical relevance [25]. In addition, femoral neck has been verified the preferred measurement site for dual energy X-ray absorptiometry measurements [26, 27]. The Hologic QDR-4500A fan-beam densitometer was utilized to acquire femoral neck BMD and BMC.

### Study covariates

Baseline variables in this study included age (years), gender, race/ethnicity, income, BMI (kg/m^2^), drinking, smoking, physical activity (PA), serum calcium (mmol/L), serum phosphorus (mmol/L), arthritis, hypertension, diabetes, cardiovascular disease and chronic kidney disease (CKD). All detailed measurement processes of variables in this study are publicly accessible at http://www.cdc.gov/nchs/nhanes/.

Races/ethnicities were classified as five groups: Mexican American, non-Hispanic Black, non-Hispanic White, other Hispanic and other race. The poverty income ratio (PIR) was utilized to assess income status. PIR < 1 was defined as poor, 1–3 as near-poor and ≥ 3 as not poor. BMI was categorized into four groups. BMI < 18.5 was considered as underweight, 18.5–24.9 as normal weight, 25–29.9 as overweight, and ≥ 30 as obese [28]. Participants whose dietary alcohol intake was more than 0 g were defined as alcohol consumers. Individuals who self-reported former or current smoking-cigarette were defined as smokers. PA was evaluated by weekly total metabolic equivalent task (MET). PA < 600 MET-mins/week was defined as low, 600-1200MET-mins/week as medium and ≥ 1200 MET-mins/week as high [29]. Cardiovascular disease included coronary heart disease, congestive heart failure, heart attack, stroke and angina. We used the Chronic Kidney Disease Epidemiology Collaboration formulate to assess glomerular filtration rate (eGFR) and CKD was defined as eGFR < 60 ml/min/1.73 m^2^ or urine albumin creatinine ratio (UACR) > 30 mg/g [30]. Diabetes was defined as self-reported diabetes, the use of diabetes medication or insulin, glycohemoglobin > 6.5%, fasting glucose ≥ 7.0 mmol/l, random blood glucose ≥ 11.1 mmol/l or 2-h oral glucose tolerance test blood glucose (mmol/l) ≥ 11.1 [31]. Hypertension was defined as systolic blood pressure ≥ 140 mmHg and/or diastolic blood pressure ≥ 90 mmHg based on the average of three measurements of all subjects’ blood pressure [32].

### Statistical analysis

All statistical analyses were performed following Centers for Disease Control and Prevention (CDC) guidelines. A suitable sample weight was utilized to calculate all statistical analyses in order that the data corresponded to the non-institutionalized civilian population. Continuous variables were expressed as the mean ± standard deviation (SD), whereas categorical variables were expressed as counts (percentages). One-way ANOVA test (for normally distributed continuous variables), Kruskal–Wallis *H*-test (for non-normally distributed continuous variables) and Chi-square test (for categorical variables) were utilized to measure differences among DII tertiles. Weighted multivariable linear regression analysis and smooth curve fittings were set up to estimate the association between DII with BMD and BMC. To further examine the covariable effect on this correlation, we employed Model 1 (unadjusted), Model 2 (age, gender and race/ethnicity were adjusted), and Model 3 (all covariates in Table 1 were adjusted). Considering BMI on bone health [33, 34], we employed the further subgroup analyses by BMI categories. *P* < 0.05 with effective confidence interval (CI) was of statistical significance. All analyses were constructed with R version 4.0.4 (http://www.R-project.org, The R foundation).

**Table 1:** Baseline characteristics of participants

	Overall	Dietary inflammatory index			
		Tertile 1 ≥ − 5.281 to ≤ 0.605	Tertile 2 > 0.605 to ≤ 2.424	Tertile 3 > 2.424 to ≤ 5.795	*P*-value
N	10,521	3508	3508	3505	
DII, mean (SD)	1.24 (0.04)	− 0.85 (0.03)	1.54 (0.01)	3.32 (0.02)	< 0.001
Age, years, mean (SD)	49.38 (0.31)	50.41 (0.44)	48.91 (0.41)	48.68 (0.40)	0.001
Serum calcium, mmol/L, mean (SD)	2.36 (0.00)	2.37 (0.00)	2.36 (0.00)	2.3 6(0.00)	< 0.001
Serum phosphorus, mmol/L, mean (SD)	1.21 (0.00)	1.21 (0.00)	1.21 (0.00)	1.21 (0.00)	0.87
Femoral neck BMD, g/cm^2^, mean (SD)	0.82 (0.00)	0.83 (0.00)	0.83 (0.00)	0.81 (0.00)	< 0.001
Femoral neck BMC, g, mean (SD)	4.37 (0.01)	4.49 (0.02)	4.40 (0.02)	4.19 (0.02)	< 0.001
**Gender, *n* (%)**					< 0.001
Female	4953 (48.76%)	1260 (37.57%)	1615 (47.80%)	2067 (62.67%)	
Male	5568 (51.24%)	2248 (62.43%)	1893 (52.20%)	1417 (37.33%)	
**Race/ethnicity, *n*(%)**					< 0.001
Mexican American	1604 (6.51%)	575 (6.80%)	542 (6.63%)	483 (6.07%)	
Non-hispanic black	1864 (8.48%)	483 (6.06%)	593 (8.22%)	785 (11.54%)	
Non-hispanic white	5479 (75.42%)	1912 (77.91%)	1830 (74.89%)	1725 (73.09%)	
Other hispanic	863 (4.11%)	274 (3.86%)	295 (4.34%)	293 (4.17%)	
Other race—including Multi-racial	711 (5.47%)	264 (5.36%)	248 (5.92%)	198 (5.13%)	
**Income, *n* (%)**					< 0.001
Poor	1742 (10.14%)	471 (7.86%)	521 (8.90%)	744 (14.08%)	
Near poor	4148 (31.73%)	1221 (26.92%)	1418 (32.39%)	1503 (36.59%)	
Not poor	4631 (58.13%)	1816 (65.21%)	1569 (58.71%)	1237 (49.33%)	
**BMI, kg/m**^**2**^**, *n* (%)**					0.004
Underweight	144 (1.37%)	42 (1.41%)	44 (1.32%)	58 (1.39%)	
Normal	3160 (30.42%)	1110 (32.51%)	1045 (29.58%)	999 (28.89%)	
Overweight	3876 (36.31%)	1333 (36.41%)	1318 (38.12%)	1218 (34.30%)	
obese	3485 (31.9%)	1065 (29.66%)	1145 (30.98%)	1267 (35.42%)	
**Smoking, *n* (%)**	4968 (46.21%)	1583(43.86%)	1667 (45.70%)	1711 (49.60%)	0.001
**Drinking, *n* (%)**	3327 (35.92%)	1373 (43.41%)	1173 (38.13%)	773 (24.88%)	< 0.001
**Physical activity, *n* (%)**					< 0.001
Low	2880 (27.92%)	820 (23.43%)	989 (29.88%)	1063 (30.94%)	
Medium	1704 (16.23%)	568 (15.69%)	571 (16.45%)	563 (16.64%)	
High	5937 (55.86%)	2120 (60.88%)	1948 (53.67%)	1858 (52.42%)	
**Comorbid disease**					
Arthritis, *n* (%)	2899 (26.85%)	942 (26.78%)	932 (24.95%)	1017 (28.87%)	0.03
Hypertension, *n* (%)	4408 (37.18%)	1407 (35.53%)	1443 (36.43%)	1545 (39.74%)	0.02
Cardiovascular disease, *n* (%)	1035 (7.8%)	298 (6.95%)	347 (7.85%)	386 (8.68%)	0.16
Diabetes, *n* (%)	2597 (19.91%)	841 (19.02%)	847 (19.81%)	905 (21.12%)	0.2
CKD, *n* (%)	1642 (12.39%)	487 (10.39%)	524 (12.12%)	631 (15.06%)	< 0.001

*DII* Dietary inflammatory index, *SD* standard deviation, *BMD* Bone mineral density, *BMC* Bone mineral content, *BMI* Body mass index, *CKD* Chronic kidney disease

## Results

### Baseline characteristics of participants

Table 1 displays the basic information characteristics based on DII tertiles. As described in Fig. 1, a total of 10,521 participants aged more than 20 years participated in this study, of whom 51.24% were males, with an average age of 49.38 ± 0.31 years. The average DII was 1.24 ± 0.04, ranging from − 5.281 (most anti-inflammatory) to 5.795 (most pro-inflammatory). The overall DII score were divided into three groups (tertile 1 = − 5.281 to 0.605, tertile 2 = 0.605–2.424, tertile 3 = 2.424–5.795). Participants in tertile 3 (more pro-inflammatory diet) presented a lower level of serum calcium, femoral neck BMD and femoral neck BMC. They were more likely to be younger, female, non-Hispanic black, near poor or poor, obese, smokers, low PA, and non-alcohol consumers compared with other tertiles (*P* < 0.05). Furthermore, participants in tertile 3 (highest DII score) had a higher incidence of arthritis, hypertension and CKD than those in the tertile 1 and 2 (*P* < 0.05). No significant differences were uncovered among the DII tertiles in serum phosphorus, cardiovascular disease and diabetes (*P* > 0.05).

### Dietary inflammation and bone health

Weighted multivariable liner regression was utilized to elucidate the relationship between DII and bone health among US adults (Tables 2 and 3). Our results revealed a negative association between DII and BMD (Model 3, *β* = − 0.004, 95% Cl − 0.005, − 0.002, *P* < 0.001) and BMC (Model 3, *β* = − 0.026, 95% Cl − 0.036, − 0.016, *P* < 0.001). This negative correlation between DII with femoral BMD and BMC became stronger after DII was grouped as tertiles. The effect size was − 0.016 for BMD in the most pro-inflammatory diet (tertile 3) (95% Cl − 0.024, − 0.008, *P* < 0.001) and − 0.111 for BMC in tertile 3 (95% Cl − 0.157, − 0.066, *P* < 0.001) in the fully adjusted model.

**Table 2:** Association between dietary inflammatory index (DII) and bone mineral density (BMD) among adults

	Model 1		Model 2		Model 3	
	*β* (95% Cl)	*P*-value	*β* (95% Cl)	*P*-value	*β* (95% Cl)	*P*-value
DII continuous	− 0.003 (− 0.005, − 0.001)	0.003	− 0.002 (− 0.004, 0.000)	0.019	− 0.004 (− 0.005, − 0.002)	< 0.001
DII tertile						
Tertile 1	Ref	Ref	Ref	Ref	Ref	Ref
Tertile 2	− 0.001 (− 0.010, 0.009)	0.887	− 0.002 (− 0.009, 0.006)	0.671	− 0.004 (− 0.011, 0.003)	0.231
Tertile 3	− 0.017 (− 0.026, − 0.007)	< 0.001	− 0.011 (− 0.019, − 0.003)	0.009	− 0.016 (− 0.024, − 0.008)	< 0.001

Model 1: no adjustment

Model 2: adjusted for age, gender and race/ethnicity

Model 3: adjusted for age, gender, race/ethnicity, serum calcium, serum phosphorus, income, BMI, smoking, drinking, physical activity, arthritis, hypertension, cardiovascular disease, diabetes, CKD

*DII* Dietary inflammatory index, *BMI* Body mass index, *CKD* Chronic kidney disease, *Cl* Confidence interval

**Table 3:** Association between dietary inflammatory index (DII) and bone mineral content (BMC) among adults

	Model 1		Model 2		Model 3	
	*β* (95% Cl)	*P*-value	*β* (95% Cl)	*P*-value	*β* (95% Cl)	*P*-value
DII continuous	− 0.063 (− 0.075, − 0.050)	< 0.001	− 0.019 (− 0.029, − 0.009)	< 0.001	− 0.026 (− 0.036, − 0.016)	< 0.001
DII tertile						
Tertile 1	Ref	Ref	Ref	Ref	Ref	Ref
Tertile 2	− 0.089 (− 0.146, − 0.032)	0.003	− 0.018 (− 0.058, 0.022)	0.369	− 0.03 (− 0.069, 0.009)	0.125
Tertile 3	− 0.295 (− 0.352, − 0.239)	< 0.001	− 0.093 (− 0.140, − 0.046)	< 0.001	− 0.111 (− 0.157, − 0.066)	< 0.001

Model 1: no adjustment

Model 2: adjusted for age, gender and race/ethnicity

Model 3: adjusted for age, gender, race/ethnicity, serum calcium, serum phosphorus, income, BMI, smoking, drinking, physical activity, arthritis, hypertension, cardiovascular disease, diabetes, CKD

*DII* Dietary inflammatory index, *BMI* Body mass index, *CKD* Chronic kidney disease, *Cl* Confidence interval

### BMI-stratified subgroup analysis

**Fig. 2:**
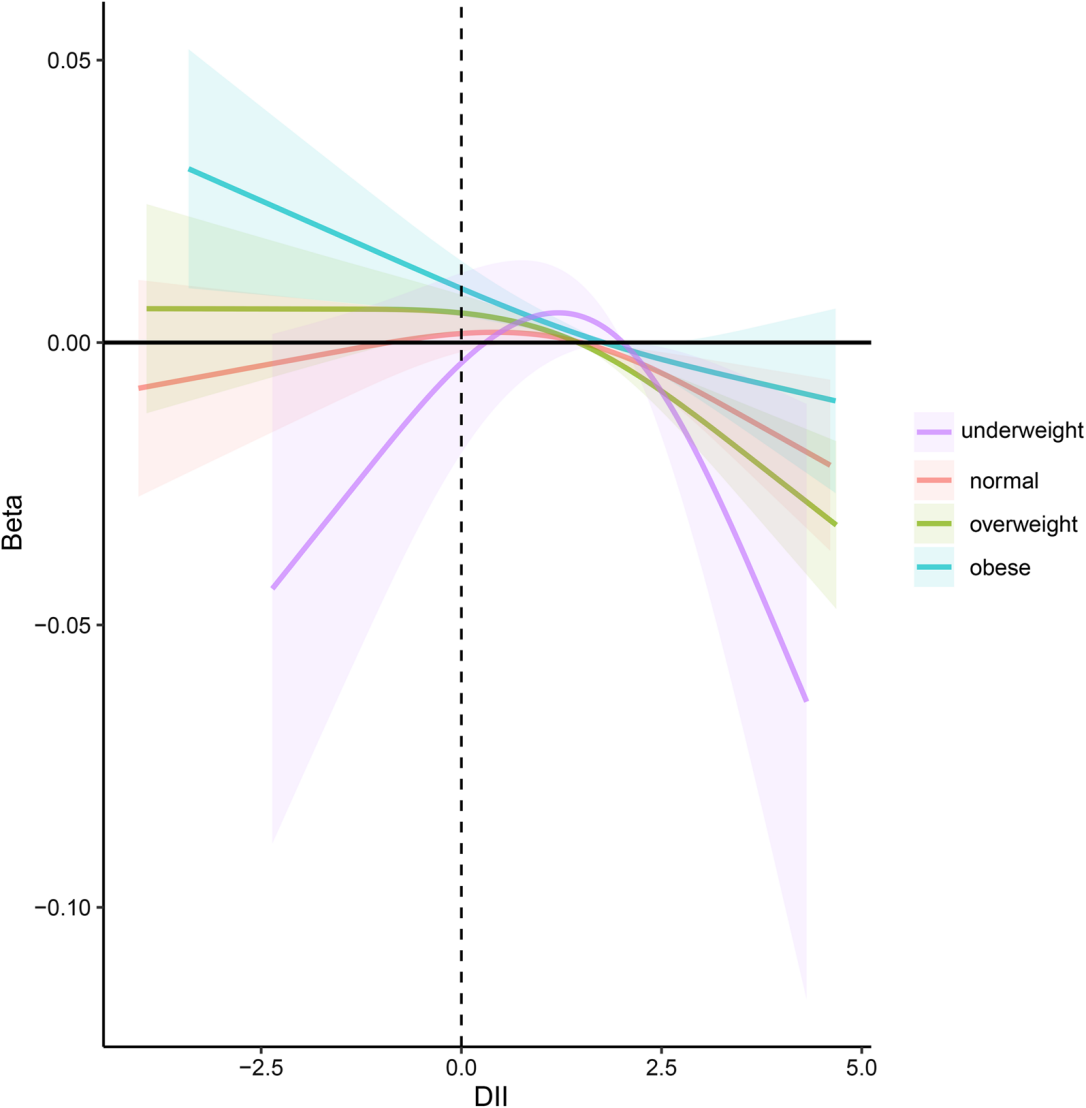
The association between DII and BMD stratified by BMI. Age, gender, race/ethnicity, serum calcium, serum phosphorus, income, smoking, drinking, physical activity, arthritis, hypertension, cardiovascular disease, diabetes and CKD were adjusted. Solid lines hazard correlation coefficient, shaded area 95% confidence interval. *DII* Dietary inflammatory index, *BMI* Body mass index, *CKD* Chronic kidney disease, *BMD* Bone mineral density

**Fig. 3:**
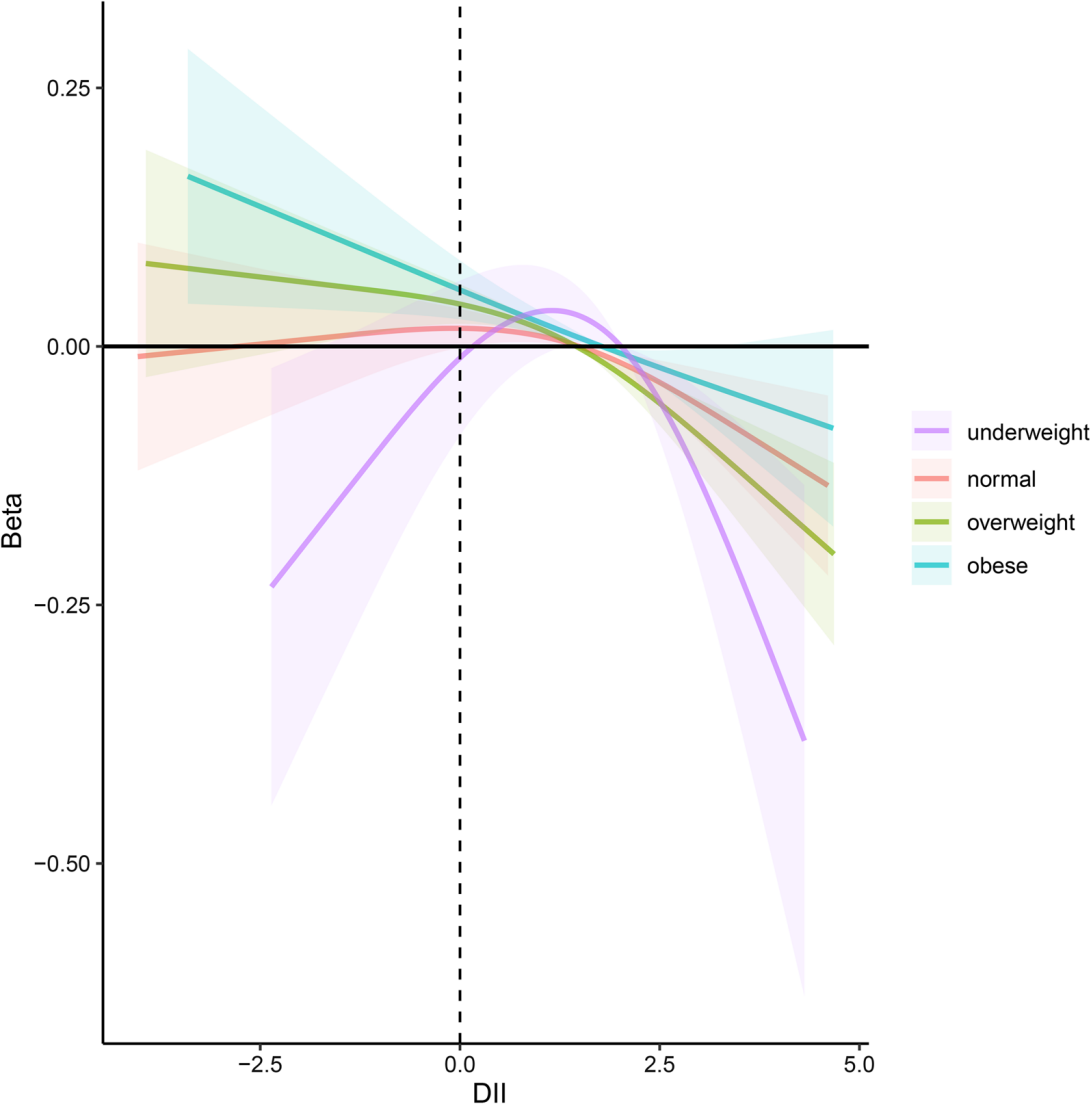
The association between DII and BMC stratified by BMI. Age, gender, race/ethnicity, serum calcium, serum phosphorus, income, smoking, drinking, physical activity, arthritis, hypertension, cardiovascular disease, diabetes and CKD were adjusted. Solid lines hazard correlation coefficient, shaded area 95% confidence interval. *DII* Dietary inflammatory index, *BMI* Body mass index, *CKD* Chronic kidney disease, *BMC* Bone mineral content

Subgroup analyses based on different BMI groups were employed to distinctly evaluate the joint influence of BMI and dietary status on the bone health. (Tables 4 and 5). Subgroup analysis revealed that higher DII was correlated with a significantly higher risk of lower BMD in the overweight (Model 3, *β* = − 0.005, 95% Cl − 0.007, − 0.02, *P* = 0.002) and obese groups (Model 3, *β* = − 0.005, 95% Cl − 0.008, − 0.002, *P* = 0.005). Furthermore, after DII was grouped as tertiles, this negative association between DII and BMD became more evident, whose effect size was -0.024 for the most pro-inflammatory diet in the overweight group (Model 3, *β* = − 0.024, 95% CI − 0.036, − 0.011, *P* < 0.001), and − 0.015 for tertile 3 in the obese group (Model 3, *β* = − 0.015,95% CI − 0.030, − 0.000, *P* = 0.049) compared with the most anti-inflammatory diet (tertile 1). Consistent with the association between DII and BMD, higher DII was also correlated with an increased risk of lower BMC in the overweight (Model 3, *β* = − 0.034, 95% Cl − 0.050, − 0.017, *P* < 0.001) and obese groups (Model 3, *β* = − 0.030, 95% Cl − 0.049, − 0.011, *P* = 0.003). After DII was divided into tertiles, this negative association remained, whose effect size was -0.058 for tertile 3 (Model3, *β* = − 0.058, 95% CI − 0.113, − 0.004, *P* = 0.042) and -0.09 for tertile 3 (Model3, *β* = − 0.09, 95% CI − 0.177, − 0.003, *P* = 0.042) in relation to tertile 1 (the most anti-inflammatory diet). Interestingly, a statistically significant positive correlation between DII and bone health was existed in DII tertile 2 (middle DII score) in the underweight group (BMD, model 3, *β* = 0.047, 95% CI 0.003, 0.090, *P* = 0.038; BMC, model 3, *β* = 0.274, 95% CI 0.073, 0.475, *P* = 0.010). To make it clear, smooth curve fittings used to characterize the joint influence of BMI and dietary status on the bone health are showed in Figs. 2 and 3.

**Table 4:** Subgroup analysis of association between dietary inflammatory index (DII) and bone mineral density (BMD) by BMI groups among adults

	Model 1		Model 2		Model 3	
	*β* (95% Cl)	*P*-value	*β* (95%Cl)	*P*-value	*β* (95% Cl)	*P*-value
*Underweight*						
DII continuous	0.001 (− 0.012,0.014)	0.859	− 0.001 (− 0.009, 0.007)	0.760	0.001 (− 0.006, 0.008)	0.792
DII tertile						
Tertile 1	Ref	Ref	Ref	Ref	Ref	Ref
Tertile 2	0.06 (− 0.008,0.128)	0.081	0.041 (− 0.010, 0.093)	0.105	0.047 (0.003, 0.090)	0.038
Tertile 3	− 0.022 (− 0.081,0.037)	0.447	− 0.023 (− 0.077, 0.030)	0.362	− 0.017 (− 0.060, 0.026)	0.415
*Normal weight*						
DII continuous	− 0.001 (− 0.005,0.003)	0.713	− 0.002 (− 0.005, 0.001)	0.152	− 0.001 (− 0.005, 0.002)	0.369
DII tertile						
Tertile 1	Ref	Ref	Ref	Ref	Ref	Ref
Tertile 2	0.013 (− 0.004,0.030)	0.124	0.005 (− 0.008, 0.018)	0.465	0.007 (− 0.006, 0.020)	0.298
Tertile 3	− 0.009 (− 0.028,0.010)	0.364	− 0.013 (− 0.027, 0.002)	0.099	− 0.009 (− 0.024, 0.007)	0.262
*Overweight*						
DII continuous	− 0.007 (− 0.010, − 0.004)	< 0.001	− 0.005 (− 0.007, − 0.002)	< 0.001	− 0.005 (− 0.007, − 0.002)	0.002
DII tertile						
Tertile 1	Ref	Ref	Ref	Ref	Ref	Ref
Tertile 2	− 0.011 (− 0.024, 0.001)	0.080	− 0.009 (− 0.021, 0.002)	0.109	− 0.009 (− 0.021, 0.002)	0.111
Tertile 3	− 0.035 (− 0.051, − 0.020)	< 0.001	− 0.025 (− 0.037, − 0.013)	< 0.001	− 0.024 (− 0.036, − 0.011)	< 0.001
*Obese*						
DII continuous	− 0.006 (− 0.010, − 0.003)	< 0.001	− 0.006 (− 0.009, − 0.002)	0.002	− 0.005 (− 0.008, − 0.002)	0.005
DII tertile						
Tertile 1	Ref	Ref	Ref	Ref	Ref	Ref
Tertile 2	− 0.009 (− 0.025, 0.007)	0.250	− 0.01 (− 0.022, 0.003)	0.129	− 0.009 (− 0.021, 0.004)	0.161
Tertile 3	− 0.02 (− 0.035, − 0.005)	0.012	− 0.017 (− 0.033, − 0.002)	0.027	− 0.015 (− 0.030, 0.000)	0.049

Model 1: no adjustment

Model 2: adjusted for age, gender and race/ethnicity

Model 3: adjusted for age, gender, race/ethnicity, serum calcium, serum phosphorus, income, smoking, drinking, physical activity, arthritis, hypertension, cardiovascular disease, diabetes, CKD

*DII* Dietary inflammatory index, *BMI* Body mass index, *CKD* chronic kidney disease, *Cl* Confidence interval

**Table 5:** Subgroup analysis of association between Dietary Inflammatory index (DII) and bone mineral content (BMC)by BMI groups among adults

	Model 1		Model 2		Model 3	
	*β* (95% Cl)	*P*-value	*β* (95% Cl)	*P*-value	*β* (95% Cl)	*P*-value
*Underweight*						
DII continuous	0.005 (− 0.069,0.079)	0.889	− 0.007 (− 0.047, 0.032)	0.702	0.002 (− 0.035, 0.038)	0.944
DII tertile						
Tertile 1	Ref	Ref	Ref	Ref	Ref	Ref
Tertile 2	0.396 (0.066,0.727)	0.021	0.252 (0.020, 0.484)	0.035	0.274 (0.073, 0.475)	0.010
Tertile 3	− 0.129 (− 0.444,0.185)	0.399	− 0.134 (− 0.376, 0.108)	0.252	− 0.107 (− 0.332, 0.118)	0.334
*Normal weight*						
DII continuous	− 0.039 (− 0.064, − 0.013)	0.003	− 0.02 (− 0.037, − 0.002)	0.026	− 0.013 (− 0.031, 0.004)	0.133
DII tertile						
Tertile 1	Ref	Ref	Ref	Ref	Ref	Ref
Tertile 2	− 0.013 (− 0.116, 0.089)	0.800	0.009 (− 0.064, 0.082)	0.815	0.026 (− 0.047, 0.098)	0.478
Tertile 3	− 0.215 (− 0.331, − 0.099)	< 0.001	− 0.1 (− 0.182, − 0.018)	0.017	− 0.07 (− 0.154, 0.014)	0.101
*Overweight*						
DII continuous	− 0.09 (− 0.111, − 0.069)	< 0.001	− 0.037 (− 0.052, − 0.021)	< 0.001	− 0.034 (− 0.050, − 0.017)	< 0.001
DII tertile						
Tertile 1	Ref	Ref	Ref	Ref	Ref	Ref
Tertile 2	− 0.148 (− 0.240, − 0.055)	0.002	− 0.066 (− 0.139, 0.006)	0.071	− 0.064 (− 0.138, 0.009)	0.083
Tertile 3	− 0.418 (− 0.517, − 0.318)	< 0.001	− 0.181 (− 0.255, − 0.108)	< 0.001	− 0.058 (− 0.113, − 0.004)	0.042
*Obese*						
DII continuous	− 0.088 (− 0.108, − 0.067)	< 0.001	− 0.035 (− 0.055, − 0.016)	< 0.001	− 0.03 (− 0.049, − 0.011)	0.003
DII tertile						
Tertile 1	Ref	Ref	Ref	Ref	Ref	Ref
Tertile 2	− 0.152 (− 0.243, − 0.060)	0.001	− 0.049 (− 0.116, 0.017)	0.144	− 0.039 (− 0.105, 0.026)	0.236
Tertile 3	− 0.333 (− 0.428, − 0.238)	< 0.001	− 0.112 (− 0.202, − 0.021)	0.016	− 0.09 (− 0.177, − 0.003)	0.042

Model 1: no adjustment

Model 2: adjusted for age, gender and race/ethnicity

Model 3: adjusted for age, gender, race/ethnicity, serum calcium, serum phosphorus, income, smoking, drinking, physical activity, arthritis, hypertension, cardiovascular disease, diabetes, CKD

*DII* Dietary inflammatory index, *BMI* Body mass index, *CKD* Chronic kidney disease, *Cl* Confidence interval

## Discussion

To our knowledge, this is the first study that assesses the relationship between DII and bone health under different levels of BMI. In this cross-sectional study that incorporated 10,521 US adults (age ≥ 20), we demonstrated that a pro-inflammatory diet was correlated with an increased risk of lower BMD and BMC only in the overweight and obese participants, while this correlation was opposite in DII tertile 2 (middle DII score) in the underweight group. Previous findings on DII and osteoporosis omitted the joint influence of BMI and dietary status on the bone health [35–38]. This gap seemed to be filled in the present study.

This is a large-scale cross-sectional study evaluating an association between dietary inflammatory potential and bone health among US adults. Emerging evidence has revealed that dietary intake plays an important role in bone health [39, 40]. A meta-analysis revealed that people who adhered to the Mediterranean dietary pattern with a high consumption of fruits, vegetables, nuts, fish and legumes and a low consumption of meats and sugars was linked with a small but important increase in BMD [39]. Another meta-analysis reported that healthy dietary patterns, high in vegetables, fruit, whole grains and long-chain *n*-3 fats and low in sugar-sweetened beverages, fruit juices, red and processed meat, was linked with decreased risk of fracture [40]. It was validated that excessive consumption of tea, alcohol and coffee increase the risk of decreased BMD and vitamin C, vitamin D, and calcium can be utilized to prevent low BMD [13]. However, in this study, participants with higher intake of anti-inflammatory diet had higher alcohol intake, which may be consistent with the amount of alcohol intake. A review noted that individuals with consumption of up to one or two standard drinks of alcohol per day were associated with higher femur neck BMD, but opposite while up to three standard drinks of alcohol per day compared to individuals with no alcohol consumption [41].

The findings of the BMI-stratified subgroup analysis revealed that the negative correlation between higher DII with increased risk of lower BMD and BMC only existed in the overweight and obese US adults, while this correlation was opposite in DII tertile 2 (middle DII score) in the underweight group. Previous studies have proved that high BMI has a positive effect on BMD in both men and women [42, 43]. The controversies between BMI and BMD are gender-specific, age-specific and different skeletal sites. A recent cohort study based on 285,643 Korean adults confirmed that low BMI was a trigger for all fractures and obesity was a trigger factor for proximal humerus fracture, but it is a protective factor for wrist fracture [44]. Further, a meta-analysis reflected that low BMI was a risk for vertebral fracture in men, but not in women [45]. Additionally, a retrospective study indicated that the relationship between the body weight and BMD was statistically significant only in obese population in elder groups in both genders [43]. However, the research about high BMI being a protective factor for fracture has been challenged [46]. A population-based prospective study based on 60,393 women indicated that obesity was a risk factor for fracture in postmenopausal women [21, 47]. Similarly, in the present study, the overweight and obese participants with higher pro-inflammatory diet had an increased risk of lower BMD and BMC. The reason could be that inflammation is related to BMI. Previous study reported that the neutrophil/lymphocyte and platelet/lymphocyte ratio and systemic immune-inflammatory index were significantly affected by BMI status [47]. With respect to underweight participants, there are commonly combined with malnutrition and dietary improvement, such as mild pro-inflammatory diet, may promote bone resorption. A prospective study of 26,318 participants revealed that risk of hip fracture in underweight participants was 45% lower for every 25 g/day protein (a pro-inflammatory food parameter) consumed [48].

Accumulating studies have reported that bone health has a close association with pro-inflammatory cytokines, including interleukin (IL)-1, tumor necrosis factor-alpha (TNF-α), IL-6, or interferon-gamma. IL-6 was essential for triggering osteoclast differentiation and activation [10]. IL-1 was also a trigger for bone resorption, accelerating bone loss in idiopathic and postmenopausal osteoporosis [9, 11]. TNF-α stimulated the inflammatory mediator nitric oxide, promoting osteoblast apoptosis [12]. Moreover, chronic inflammation maintained by diet was also a trigger for osteoporosis. On the other hand, anti-inflammatory nutrition was found to correlate with bone health. A meta-analysis showed that zinc supplementation/intake may help to improve the levels of serum osteocalcin, serum alkaline phosphatase and femoral neck BMD [14]. As for *n*-3 polyunsaturated fatty acid (PUFA), an anti-inflammatory food parameter, a systematic review and meta-analysis reported that *n*-3 PUFA might exert a beneficial effect on bone health [15] by inhibiting oxidative stress [4], down-regulating peroxisome proliferator-activated receptor gamma and enhancing osteoblastic activity [49]. Another meta-analysis summarized that vitamin C intake decreased the risk of bone mineral density loss [50].

This study has some limitations. Firstly, the cross-sectional study was designed to examine the correlation between DII and bone health instead of the causal references. Secondly, dietary habit recall related bias is inevitable although the NHANES design utilized sampling weight and multiple-pass method to ensure precise of dietary intake and subjects who had both two valid 24-h dietary recalls were included in this study. Thirdly, further studies about whether the negative association between DII and bone heath is suitable for young people or different national groups should be performed. Fourthly, more clinical information including the use of drugs should be subsumed to assess this association. Finally, we did not assess the level of fat mass and lean soft tissue in this study. Lean soft tissue is an important determinant of bone health and BMI is not capable of differentiating between fat mass and lean soft tissue. Further studies including the level of fat mass and lean soft tissue would provide a better understanding of the relation between DII and bone health.

## Conclusion

In this cross-sectional study with 10,521 US adults, higher intake of pro-inflammatory diet was correlated with an increased risk of lower BMD and BMC only in overweight and obese participants, while this correlation was opposite in DII tertile 2 (middle DII score) in the underweight group. Further studies for validating the causal references between DII and bone health are warranted.

## Data Availability

This data are publicly available at NHANES website (http://www.cdc.gov/nchs/nhanes).
